# Increased clonal dissemination of OXA-232-producing ST15 *Klebsiella pneumoniae* in Zhejiang, China from 2018 to 2021

**DOI:** 10.1186/s40249-023-01051-w

**Published:** 2023-03-22

**Authors:** Yanyan Zhang, Xuemei Yang, Congcong Liu, Ling Huang, Lingbin Shu, Qiaoling Sun, Hongwei Zhou, Yonglu Huang, Chang Cai, Xiaoyan Wu, Sheng Chen, Rong Zhang

**Affiliations:** 1 Department of Clinical Laboratory, Second Affiliated Hospital, Zhejiang University School of Medicine, Hangzhou, China; 2 Department of Infectious Diseases and Public Health, Jockey Club College of Veterinary Medicine and Life Sciences, City University of Hong Kong, Kowloon, Hong Kong China; 3 Department of Clinical Laboratory Medicine, Maternal and Child Health Hospital of Yuhang District, Hangzhou, China; 4 College of Animal Science and Technology, Zhejiang Agricultural and Forestry University, Hangzhou, China; 5 The Clinical Laboratory, Jiaxing Second Hospital, Jiaxing, China

**Keywords:** *Klebsiella pneumoniae*, *Escherichia coli*, OXA-232, ColKP3, Clonal dissemination

## Abstract

**Background:**

OXA-232-producing *Klebsiella pneumoniae* was first identified in China in 2016, and its clonal transmission was reported in 2019. However, there are no prevalence and genotypic surveillance data available for OXA-232 in China. Therefore, we investigated the trends and characteristics of OXA-232 type carbapenemase in Zhejiang Province, China from 2018 to 2021.

**Methods:**

A total of 3278 samples from 1666 patients in the intensive care units were collected from hospitals in Zhejiang Province from 2018 to 2021. Carbapenem-resistant isolates were initially selected by China Blue agar plates supplemented with 0.3 μg/ml meropenem, and further analyzed by matrix-assisted laser desorption/ionization-time-of-flight mass spectrometry identification, immune colloidal gold technique, conjugation experiment, antimicrobial susceptibility testing and whole genome sequencing.

**Results:**

A total of 79 OXA-producing strains were recovered, with the prevalence increased from 1.8% [95% confidence interval (*CI*): 0.7–3.7%] in 2018 to 6.0% (95% *CI*: 4.4–7.9%) in 2021. Seventy-eight strains produced OXA-232 and one produced OXA-181. The *bla*_OXA-232_ gene in all strains was located in a 6141-bp ColKP3-type non-conjugative plasmid and the *bla*_OXA-181_ gene was located in a 51,391-bp ColKP3/IncX3-type non-conjugative plasmid. The *bla*_OXA-232_-producing *K. pneumoniae* was dominated (75/76) by isolates of sequence type 15 (ST15) that differed by less than 80 SNPs. All OXA-producing strains (100%, 95% *CI*: 95.4–100.0%) were multidrug-resistant.

**Conclusions:**

From 2018 to 2021, OXA-232 is the most prevalent OXA-48-like derivative in Zhejiang Province, and ST15 *K. pneumoniae* isolates belonging to the same clone are the major carriers. The transmission of ColKP3-type plasmid to *E. coli* highlighted that understanding the transmission mechanism is of great importance to delay or arrest the propagation of OXA-232 to other species.

**Supplementary Information:**

The online version contains supplementary material available at 10.1186/s40249-023-01051-w.

## Background

Antibiotic resistance has become one of the major stumbling blocks on the road to human health. It is worth noting that carbapenem-resistant *Enterobacterales* (CRE) played a significant role in this challenge. The oxacillinase OXA-48 was first identified in 2004 from a clinically collected *Klebsiella pneumoniae* isolate in Turkey [1]. Since then, OXA-48-like enzymes have emerged and spread rapidly around the world and remain endemic in the Mediterranean Region, such as Turkey, Lebanon, and Egypt [29]. OXA-181, which contains four amino acid substitutions compared to OXA-48, was first discovered in India and is prevalent in the Indian subcontinent [3–6]. OXA-232 was first reported in 2013 from *K. pneumoniae* and *Escherichia coli* obtained from three French patients with a travel history to India. Such carbapenemases have also caused nosocomial outbreaks in different countries [7–11]. OXA-181 and OXA-232 are the most common OXA-48 derivatives, differing by only one amino acid substitution [7, 12].

Bacterial strains carrying the *bla*_OXA-232_ gene were first reported in China in 2016. The gene is located on a ColKP3-type nonconjugative plasmid (also known as ColE-type) from sequence type (ST) 15 *K. pneumoniae*, and the subsequent clonal dissemination was reported in 2018 [8, 9]. The ColKP3-type plasmids have been identified in many countries, such as ST14 *K. pneumoniae* in South Korea, ST14, ST231, ST395 *K. pneumoniae* and ST167 *E. coli* in Canada, ST16 *K. pneumoniae* in Italy [10, 13, 14]. OXA-181-producing *E. coli* and *K. pneumoniae* isolates had been reported sporadically in China with all *bla*_OXA-181_ genes located on the IncX3-type plasmid [15–18]. Moreover, bacteria co-producing OXA-48 family and other carbapenemases such as *K. pneumoniae* carbapenemase (KPC) and the New Delhi metallo-β-lactamase (NDM) were intermittently reported worldwide [19–21]. In 2017, OXA-232 associating with ST147 *K. pneumoniae* was reported in Tunisia with the coproduction of extended-spectrum β-lactamase (ESBL) CTX-M-15 [22]. The co-production of OXA-232 with various key enzymes could increase the MICs to carbapenems and cause resistance to other common antibiotics such as extended-spectrum cephalosporins, ceftazidime/avibactam, and therefore pose a looming threat to human health. So far, only a limited number of nosocomial OXA-48-like carbapenemases reports were published in Zhejiang Province, long-term surveillance focusing on its prevalence and molecular characteristics is therefore urgently needed to provide a reference to making future surveillance strategy [8, 18]. Herein, we designed this study to identify the prevalence and molecular characteristics of *bla*_OXA_-producing CRE in Zhejiang Province from 2018 to 2021.

## Materials and methods

### Sample collection

This experiment was designed to investigate the prevalent trends and characteristics of OXA type carbapenemase in Zhejiang, China, from 2018 to 2021. A total of 2512 respiratory and rectal swabs from 1283 ICU patients were collected in six regions of Zhejiang Province, China, from March 2020 to June 2021. 1576 respiratory and rectal swabs from 788 ICU patients were collected from six cities in Zhejiang Province, including Hangzhou, Taizhou, Jinhua, Wenzhou, Lishui, and Quzhou in 2021. 936 respiratory and rectal swabs of 495 patients were collected from eight cities in Zhejiang province, including Hangzhou, Taizhou, Jiaxing, Huzhou, Shaoxing, Zhoushan, Ningbo, and Wenzhou in 2020 (Additional file 2: Table S1). In addition, ten OXA-producing *Enterobacterales* were recovered from 766 respiratory and rectal swabs of 383 ICU patients from six cities in Zhejiang Province in 2018 were also included in this study [8]. All samples were collected from patients who signed informed consent form when they were completing the admission procedures.

### Bacterial cultivation and resistance genes primary screening

Firstly, the respiratory and rectal swabs were incubated overnight at 37 °C in Luria–Bertani (LB) broth (Oxoid, UK) for enrichment. The broth suspensions were then inoculated onto China Blue agar plates containing 0.3 μg/ml meropenem and incubated overnight in 37 °C. All colonies were subjected to identification using the matrix-assisted laser desorption/ionization-time-of-flight mass spectrometry (MALDI-TOF MS) (Fosun Diagnostics Co., Ltd, Shanghai, China). The presences of carbapenemase genes were screened by NG-Test^®^ CARBA 5 (Zhongshengzhongjie Bio-Technology Co., Ltd., Changsha, China).

### Antimicrobial susceptibility testing

Antimicrobial susceptibility testing was carried out by the broth microdilution method. Antibiotics in the antimicrobial susceptibility testing comprises imipenem, meropenem, ertapenem, cefmetazole, ceftazidime, cefotaxime, piperacillin/tazobactam, cefopcrazone/sulbactam, ceftazidime/avibactam, cefepime, Polymyxin B, tigecycline, ciprofloxacin, amikacin, aztreonam. The results for tigecycline were interpreted based on the European committee on antimicrobial susceptibility testing (EUCAST) and the others were interpreted according to Clinical and Laboratory Standards Institute (CLSI) [23, 24].

### Conjugation

The conjugation experiment was performed by the filter-mating method using rifampin-resistant *E. coli* EC600 as recipient as previously reported [25]. In short, the donor strain and recipient strain *E. coli* EC600 were cultivated at 37 °C in LB broth for four hours, respectively, and then co-incubated overnight on a membrane placed on Columbia Blood Agar plate. Membranes were swirled in Luria–Bertani (LB) broth, the suspension cultured overnight at 37 °C on Mueller–Hinton (MH) agar plates containing 1 μg/ml meropenem and 600 μg/ml rifampin. Finally, the transconjugants were verified by THE MALDI-TOF MS and Polymerase Chain Reaction [26, 27].

### Whole genome sequencing and bioinformatics analysis

Genomic DNA of all the OXA-producing isolates were extracted using the PureLink Genomic DNA Mini Kit (Invitrogen, Carlsbad, CA, USA) following manufacturer’s instructions and submitted for whole genome sequencing using the Illumina HiSeq X Ten platform (Novogene, Beijing, China). The Illumina reads were de novo assembled using SPAdes Genome Assembler version 3.15.1 [28]. Assembled draft genome sequences were annotated with Prokka version 1.14.5 [29]. Single nucleotide polymorphisms (SNPs) were identified via mapping of Illumina raw reads to genome of strain K210003 as reference. An alignment of core SNPs was produced using Snippy and used to build a high-resolution phylogeny [30]. Lineages were defined based on patristic distances in the maximum-likelihood (ML) tree using IQ-Tree [31]. The output tree was then merged to attain a dated tree with the online TreeAnnotator software iTOL [32]. Bioinformatics analysis including species identity, multilocus sequence typing (MLST) and identification of antimicrobial resistance genes (ARGs) of *K. pneumoniae* was conducted with Kleborate [33]. Capsular typing on the assembled sequences was performed using Kaptiveg [34]. Multilocus sequence typing of *E. coli* were confirmed on the center for genomic epidemiology platform [35]. Plasmid replicons were identified by PlasmidFinder [36].

To obtain the complete genome of strains K210049 and K210065, genomic DNA of these two strains were also subjected to the long-read Oxford Nanopore Technologies MinION platform (Oxford Nanopore Technologies, Abingdon, United Kingdom) after treated with supplementary sequencing kit (Oxford Nanopore Technologies, Abingdon, United Kingdom). Both short and long reads were de novo hybrid assembled using Unicycler version 0.4.8 [37]. Alignment of plasmids with similar structures were generated by Easyfig_win_2.1 and BLAST Ring Image Generator (BRIG) version 0.95.22 [38, 39].

### Data analysis

Data was organized and analyzed by Statistical Products and Services Solutions (SPSS) software (IBM, USA), and 95% confidence intervals (*CI*s) were calculated by the exact binomial method.

## Results

### Prevalence of OXA-carrying patients

Whole genome sequencing results indicated that ten and 59 OXA-producing enterobacterial isolates were collected in 2020 and 2021 respectively. Ten OXA-producing *K. pneumoniae* strains from 383 patients reported in 2018 were also included for comparison [8]. The prevalence of OXA-type carbapenemase was 6.0% (95% *CI*: 4.4–7.9%) in 2021, which was relatively higher than 1.8% (95% *CI*: 0.8–3.4%) in 2020 and 1.8% (95% *CI*: 0.7–3.7%) in 2018.

### Antimicrobial susceptibility profiles

Antimicrobial susceptibility testing results indicated that all the OXA-producing strains were multiple drug-resistant, with all strains being resistant to sulbactam/cefopcrazone. The numbers of strains resistant to imipenem, meropenem, ertapenem, cefmetazole, piperacillin/tazobactam, polymyxin B, tigecycline, amikacin were 22, 57, 77, 14, 77, 6, 32, 74, respectively. Number of strains resistant to ceftazidime, cefotaxime, cefepime, ciprofloxacin, aztreonam were 76. All strains were susceptible to ceftazidime/avibactam expect K210284, which co-produced NDM-1 (Additional file 4: Table S3).

### Genomic characteristics of OXA-producing strains

**Fig. 1:**
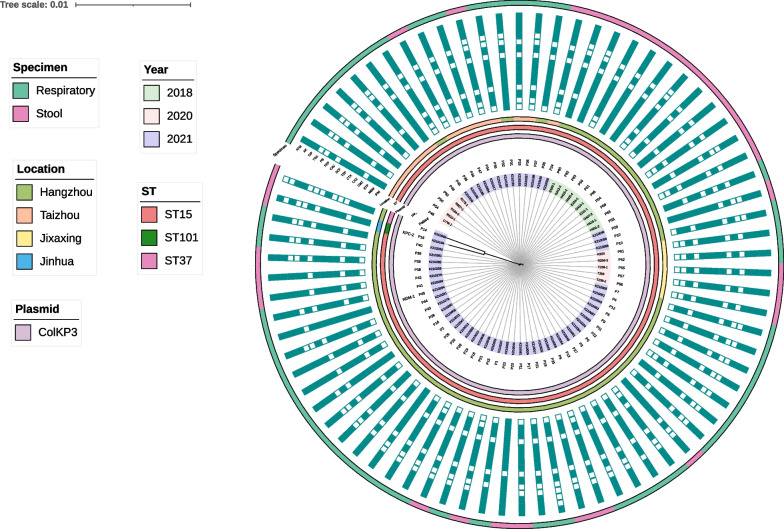
Phenotypic, genetic and distribution characteristics of OXA-producing *Klebsiella pneumoniae*. *IPM* imipenem, *MEM* meropenem, *ETP* ertapenem, *CMZ* cefmetazole, *CAZ* ceftazidime, *CTX* cefotaxime, *TZP* piperacillin/tazobactam, *SCF* sulbactam/cefopcrazone, *CAV* ceftazidime/Avibactam, *FEP* cefepime, *PB* polymyxin B, *TGC* tigecycline, *CIP* ciprofloxacin, *AK* amikacin, *ATM* aztreonam

All 79 OXA-producing isolates were subjected to WGS, and 77 were identified to be *K. pneumoniae* and two were *E. coli* (Table 1). The *bla*_OXA_ genes were found to be *bla*_OXA-232_ (78/79) and *bla*_OXA-181_ (1/79). Among the 77 OXA-producing *K. pneumoniae* strains, 75 belonged to ST15/KL112, one belonged to ST37/KL118 and one belonged to ST101/KL106. The ST101 strain K210184, which was recovered from a patient (P16) when he was hospitalized in Jinhua city, also harbored the *bla*_KPC-2_ gene except for the *bla*_OXA-232_ gene (Fig. 1). In addition, ST15 strain K210005 which harbored the *bla*_OXA-232_ gene and ST11 strain K210004 which harbored the *bla*_KPC-2_ gene (not included in this study) were also separated from the same patient during his earlier hospitalization in Hangzhou city. The OXA-232 positive strain K210284 recovered from patient P45 also carried the *bla*_NDM-1_ gene. While the ST37 strain K210065 collected in 2021 harbored the *bla*_OXA-181_ gene other than the *bla*_OXA-232_ gene (Fig. 1). The two OXA-producing *E. coli* strains belonged to ST38 and ST39, respectively, and were collected from patients P1 and P2 in the same ward. Notably, the OXA-232-producing *K. pneumoniae* strains K210037 and K210022 were isolated from patients P1 and P2, respectively, indicating potential transmission of the *bla*_OXA-232_-carrying determinants from *K. pneumoniae* to *E. coli* (Fig. 1).

As the ten *K. pneumoniae* stains isolated in 2018 exhibited only a few SNPs and belonged to a single clone, we performed pairwise SNP analysis of all the ST15 *K. pneumoniae* stains. The strains isolated in 2020 from Jiaxing and Taizhou differed from the isolates in 2018 with < 50 SNPs (Additional file 3: Table S2). The nine isolates from Taizhou in 2021 together with three isolates from Hangzhou exhibited SNPs < 8. 31 strains isolated from Hangzhou in 2021 exhibited SNPs < 10. Another nine strains isolated in this region exhibited SNPs < 4. These two clones differed with < 10 SNPs. The ST15 OXA-producing *K. pneumoniae* stains have circulated in Zhejiang Province in the past few years as all the ST15 *K. pneumoniae* stains differed with SNPs < 80. The ST15 remained the most prevalent OXA-232 *K. pneumoniae*. OXA-232-positive *E. coli* and ST101 *K. pneumoniae* only started emerging in 2021.

### Characteristics of plasmid genes

**Fig. 2:**
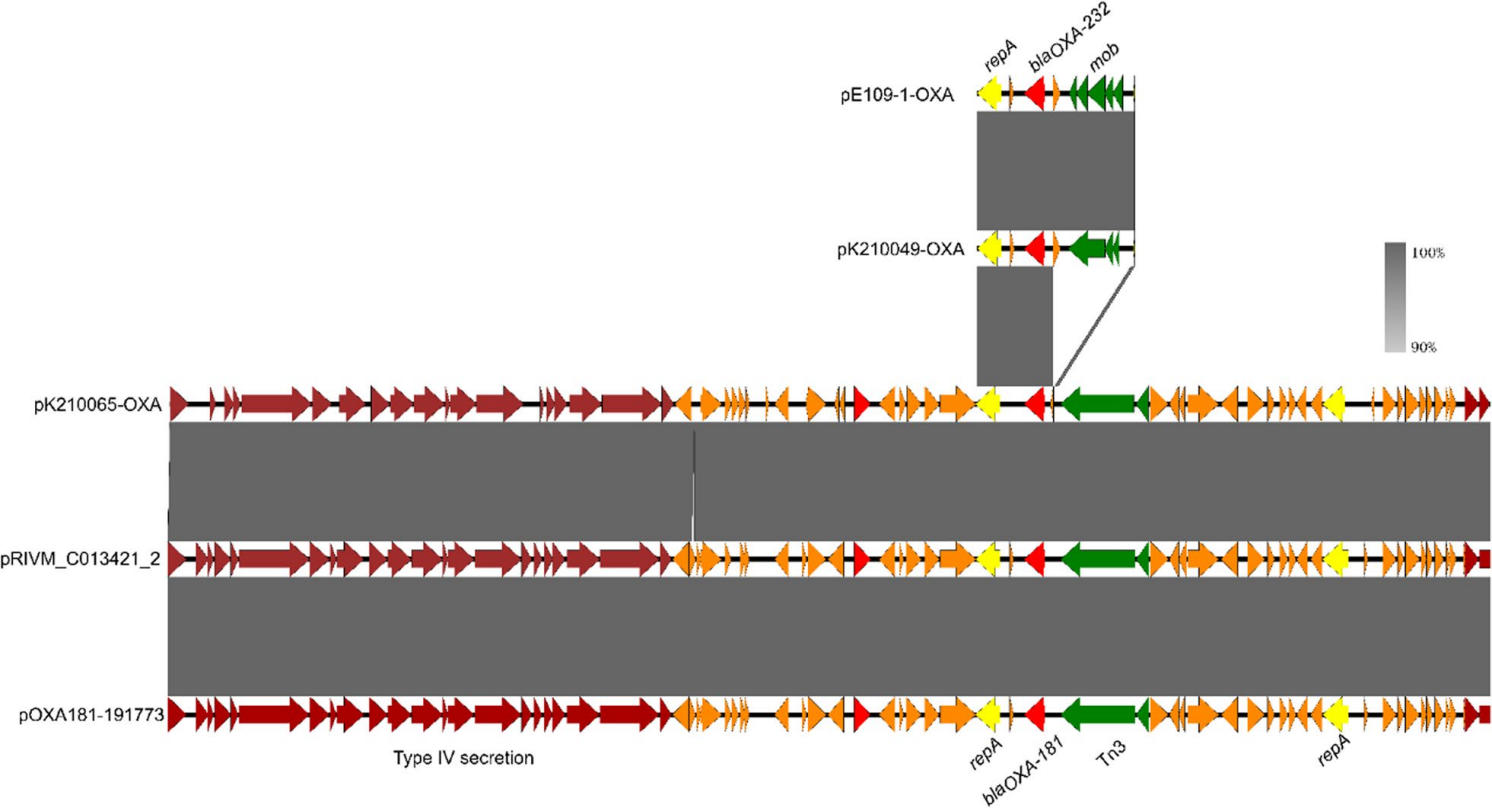
Genetic environments of *bla*_OXA-181_ and *bla*_OXA-232_

The complete sequences of the chromosome and all plasmids of *K. pneumoniae* strain K210049 isolated in 2021, were obtained. *K. pneumoniae* strain K210049 harbored a 5,340,981-bp chromosome and nine plasmids with size of 177,848-, 138,444-, 128,536-, 9730-, 6141-, 5640-, 4510-, 3770-, 3559-bp, respectively. The plasmidome of strain K210049 was identical to *K. pneumoniae* stain E109-1 which was isolated in 2018. *bla*_OXA-232_ gene located in the 6141-bp ColKP3-type plasmid, was designated as pK210049-OXA. Plasmid pK210049-OXA was 100% identical to the 6.1-kb *bla*_OXA-232_-bearing plasmid pE109-1-OXA isolated from *K. pneumoniae* strain E109-1, with 100% coverage (Fig. 2). And the *bla*_OXA-232_ gene in all the strains located in the ColKP3-type plasmid (Fig. 1 Additional file 1: Figure S1). However, the conjugation experiment showed that the ColKP3-type plasmid was nonconjugative. Both the *bla*_KPC_-_2_- and *bla*_NDM_-_1_-carrying plasmids in strains K210184 and K210284, respectively, could be transferred to *E. coli* strain EC600 by conjugation, though the antimicrobial susceptibility of the transconjugants exhibited slight differences compared to the donor strains (Additional file 4: Table S3).

The complete sequences of the chromosome and all plasmids, of the *bla*_OXA-181_-harboring *K. pneumoniae* strain K210065 isolated in 2021, were obtained. *K. pneumoniae* K210065 harbored a 5,254,313-bp chromosome and three plasmids with size of 102,145-, 51,391-, 3270-bp, respectively. The *bla*_OXA-181_ gene located in the 51,391-bp ColKP3/IncX3-type plasmid, designated as pK210065-OXA (Fig. 2). Plasmid pK210065-OXA was 100% identical to the 51-kb *bla*_OXA-181_-bearing plasmid pOXA181-191773 (GenBank: CP080367.1) isolated from a *K. pneumoniae* strain and plasmid pRIVM_C013421_2 (GenBank: CP068328.1) isolated from an *E. coli* strain, with 100% coverage (Fig. 2).

## Discussion

Antimicrobial resistance has become one of the major global concerns, and carbapenems have become antibiotics of last resort. OXA-48-like enzymes possessed a stronger ability to hydrolyze oxacillin but had low activity against carbapenems and is therefore known as oxacillinases [40]. In the current study, numerous OXA-positive strains possess a similar carbapenem resistance profile, only resistant to ertapenem but susceptible to meropenem and imipenem, known as ‘the phantom menace’ in the literature [41]. Previous studies of OXA-232 only described its genetic characteristics but did not describe its prevalent situation in Zhejiang hospitals [8]. Our study collected samples from 2018 to 2021 and described both prevalence and genetic characteristics of OXA in hospitals in Zhejiang Province.

Two clones from Hangzhou in 2021 differed with < 10 SNPs, suggesting that they might originate from a single clone. The dominant clone transmission and the increased prevalence of OXA suggest that close monitoring of OXA is needed to curtail CRE spread and thus reduce the incidence of disease. Moreover, our study has found that OXA-232 was reported in more cities’ hospitals in Zhejiang in recent years than before, which indicated its wide transmission trend. So continuous surveillance was strongly recommended to minimize the problems associated with oxacillinases.

The ColKP3-type nonconjugative plasmid is approximately 6 kb in length harbored by all strains collected in our study. To date, only ST15 *K. pneumoniae* had been reported to contain the *bla*_OXA-232_ gene situated within the OXA-232 plasmid in China [8, 9, 18], ST101 *K. pneumoniae* and two *E. coli* strains were shown to produce OXA-232 in our study. Additionally, the presence of OXA-232-positive *E. coli* and *K. pneumoniae* in the same patient reflects the horizontal transfer of ColKP3-type plasmid. However, this small plasmid does not contain genes sufficient for self-transfer. We speculate that it could transmit with the help of other plasmids such as pKP3-A. Further research is needed to investigate the transmissible mechanism of ColKP3 type plasmid to prevent the further transmission to other species.

One of the limitations of our study was that we cannot apply random sampling given the importance of consent. The hospitals that we selected were only the ones agreed to join this study. Samples from different Provinces are highly recommended to be collected in further studies to reflect the prevalence of OXA in China.

Our study highlighted the importance of the combined antimicrobic susceptibility to avoid potential threats to patients, especially to those in ICU, and to reduce the spreading due to under detection.

## Conclusions

From 2018 to 2021, OXA-232 is the most prevalent OXA-48-like derivative in Zhejiang Province, and ST15 *K. pneumoniae* isolates belonging to the same clone are the major carriers. The transmission of ColKP3-type plasmid to *E. coli* highlighted that understanding the transmission mechanism is of great importance to delay or arrest the propagation of OXA-232 to other species.

**Table 1:** Distribution characteristics of OXA-producing strains

Year	Sample size	OXA-positive Isolates (Prevalence)	Species
2021	788	59 (6.0%)	*E. coli *(2); *K. pneumoniae *(57)
2020	495	10 (1.8%)	*K. pneumoniae*
2018	383	10 (1.8%)	*K. pneumoniae*

## Supplementary Information

**Additional file 1: Figure S1.** Alignment of ColKP3-type plasmid.

**Additional file 2: Table S1.** Sample Collection information in different regions of Zhejiang Province.

**Additional file 3: Table S2.** SNP analysis of all ST15 *K. pneumoniae* stains.

**Additional file 4: Table S3.** Antimicrobial susceptibility profiles of OXA-48-like-producing strains and transconjugants.

## Data Availability

Genome sequences of all strains in 2020 and 2021 have been deposited in the NCBI database under BioProject accession numbers PRJNA801358. The BioProject accession numbers for the genome sequences of the 2018 strains are PRJNA484079 and PRJNA484098.

## Abbreviations

ICU: Intensive care unit
MALDI-TOF MS: Matrix-assisted laser desorption/ionization-time-of-flight mass spectrometry
WGS: Whole genome sequencing
ST: Sequence type
CRE: Carbapenem-resistant *Enterobacterales*
KPC: *K**.** pneumoniae* Carbapenemase
NDM: New Delhi metallo-β-lactamase
ESBL: Extended-spectrum β-lactamase
LB: Luria–Bertani
EUCAST: European committee on antimicrobial susceptibility testing
CLSI: Clinical and Laboratory Standards Institute
MH: Mueller–Hinton
SNP: Single nucleotide polymorphisms
ML tree: Maximum-likelihood tree
MLST: Multilocus sequence typing
ARGs: Antimicrobial resistance genes
BRIG: BLAST Ring Image Generator
SPSS: Statistical Products and Services Solutions
95% *CI*: 95% Confidence interval
IPM: Imipenem
MEM: Meropenem
ETP: Ertapenem
CMZ: Cefmetazole
CAZ: Ceftazidime
CTX: Cefotaxime
TZP: Piperacillin/tazobactam
SCF: Sulbactam/cefopcrazone
CAV: Ceftazidime/avibactam
FEP: Cefepime
PB: Polymyxin B
TGC: Tigecycline
CIP: Ciprofloxacin
AK: Amikacin
ATM: Aztreonam
