# Supplementary figures and images for: Increased clonal dissemination of OXA-232-producing ST15 Klebsiella pneumoniae in Zhejiang, China from 2018 to 2021
Source: Infect Dis Poverty. 2023 Mar 22;12:25. doi: 10.1186/s40249-023-01051-w (PMC10031881; doi:10.1186/s40249-023-01051-w)

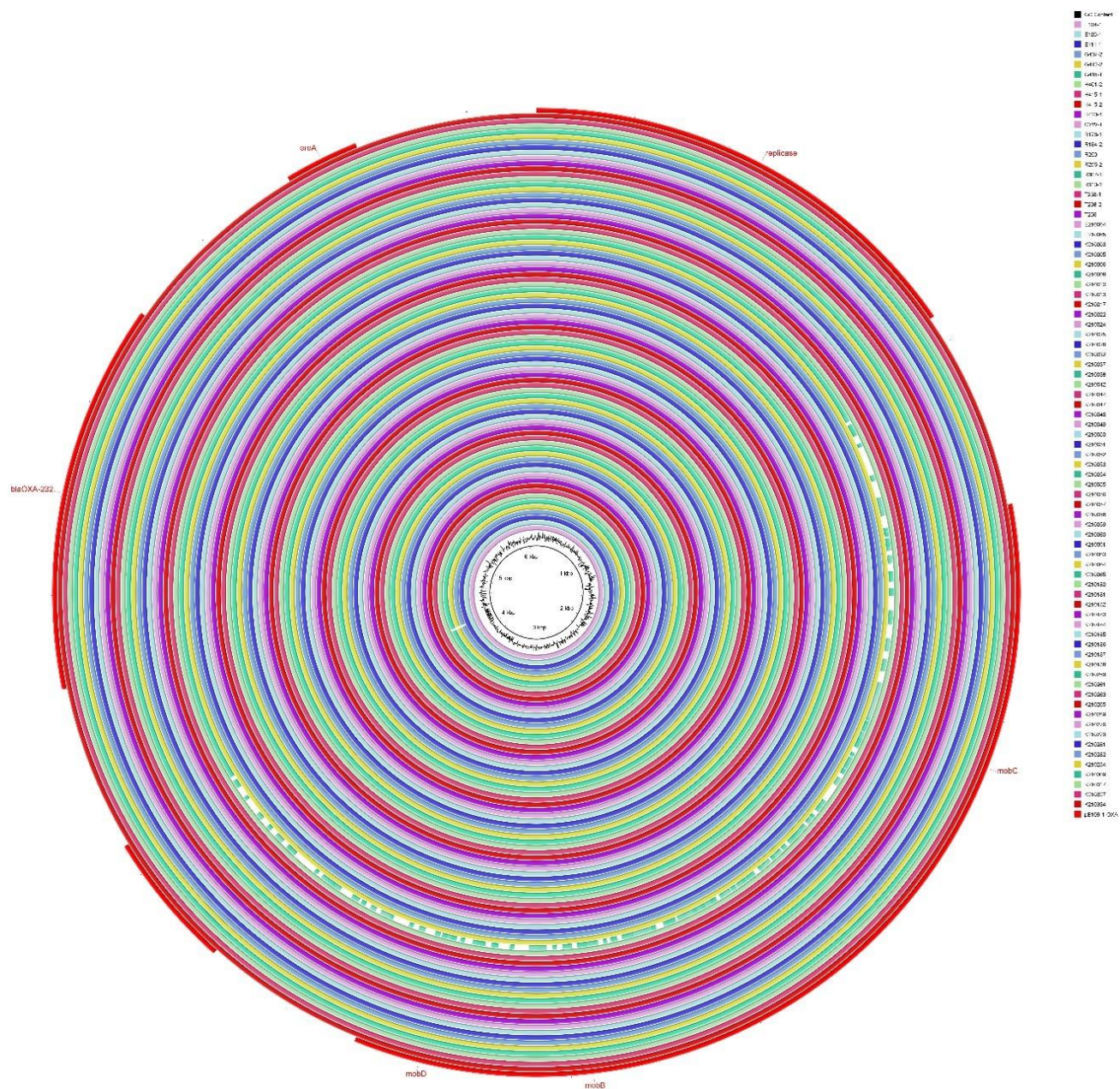

Figure S1 Alignment of ColKP3-type plasmid.

Supplement: Supplementary file 1 — Additional file 1: Figure S1. Alignment of ColKP3-type plasmid. [file 40249_2023_1051_MOESM1_ESM.pdf]
