# Supplementary material for: Increased clonal dissemination of OXA-232-producing ST15 Klebsiella pneumoniae in Zhejiang, China from 2018 to 2021
Source: Infect Dis Poverty. 2023 Mar 22;12:25. doi: 10.1186/s40249-023-01051-w (PMC10031881; doi:10.1186/s40249-023-01051-w)
Supplement: Supplementary file 2 — Additional file 2: Table S1. Sample Collection information in different regions of Zhejiang Province. [file 40249_2023_1051_MOESM2_ESM.docx]

| Year | Region | Sample size | OXA-Postive Isolates | Number of positives | OXA-KPC Postive Isolates | Number of positives | OXA-NDM Postive Isolates | Number of positives | OXA prevalence(%) |
| --- | --- | --- | --- | --- | --- | --- | --- | --- | --- |
| 2021 | Hangzhou | 542 | *E.coli* | 2 | */* | 0 | *K.pneumoniae* | 1 | 5.96% (47/788) |
|  |  |  | *K.pneumoniae* | 46 |  |  |  |  |  |
|  | Taizhou | 42 | *K.pneumoniae* | 9 | / | 0 | / | 0 |  |
|  | Jinhua | 134 | / | 0 | *K.pneumoniae* | 1 | / | 0 |  |
|  | Wenzhou | 31 | / | 0 | / | 0 | / | 0 |  |
|  | Lishui | 27 | / | 0 | / | 0 | / | 0 |  |
|  | Quzhou | 12 | / | 0 | / | 0 | / | 0 |  |
| 2020 | Taizhou | 72 | *K.pneumoniae* | 5 | / | 0 | / | 0 | 1.82% (9/495) |
|  | Jiaxing | 47 | *K.pneumoniae* | 5 | / | 0 | / | 0 |  |
|  | Hangzhou | 132 | / | 0 | / | 0 | / | 0 |  |
|  | Huzhou | 14 | / | 0 | / | 0 | / | 0 |  |
|  | Ningbo | 75 | / | 0 | / | 0 | / | 0 |  |
|  | Shaoxing | 44 | / | 0 | / | 0 | / | 0 |  |
|  | Wenzhou | 91 | / | 0 | / | 0 | / | 0 |  |
|  | Zhoushan | 20 | / | 0 | / | 0 | / | 0 |  |
| 2018 | Hangzhou | 64 | *K.pneumoniae* | 10 | / | 0 | / | 0 | 1.83% (7/383) |
|  | Wenzhou | 97 | / | 0 | / | 0 | / | 0 |  |
|  | Ningbo | 50 | / | 0 | / | 0 | / | 0 |  |
|  | Lishui | 57 | / | 0 | / | 0 | / | 0 |  |
|  | Jiaxing | 51 | / | 0 | / | 0 | / | 0 |  |
|  | Huzhou | 5 | / | 0 | / | 0 | / | 0 |  |
|  | Shaoxing | 59 | / | 0 | / | 0 | / | 0 |  |

Table. S1 Sample Collection information in different regions of Zhejiang Province.
