# Supplementary material for: Increased clonal dissemination of OXA-232-producing ST15 Klebsiella pneumoniae in Zhejiang, China from 2018 to 2021
Source: Infect Dis Poverty. 2023 Mar 22;12:25. doi: 10.1186/s40249-023-01051-w (PMC10031881; doi:10.1186/s40249-023-01051-w)
Supplement: Supplementary file 4 — Additional file 4: Table S3. Antimicrobial susceptibility profiles of OXA-48-like-producing strains and transconjugants. [file 40249_2023_1051_MOESM4_ESM.docx]

| Isolates | species | Bla_Carb | MIC (mg/L) | | | | | | | | | | | | | | |
| --- | --- | --- | --- | --- | --- | --- | --- | --- | --- | --- | --- | --- | --- | --- | --- | --- | --- |
|  |  |  | IPM | MEM | ETP | CMZ | CAZ | CTX | TZP | SCF | CAV | FEP | PB | TGC | CIP | AK | ATM |
| H401-2 | *K. pneumoniae* | OXA-232 | ≦1 | 2 | 8 | 16 | 64 | >128 | >256/4 | 256/128 | ≦0.5/4 | >64 | ≦0.5 | 1 | >32 | >128 | >128 |
| G407-2 | *K. pneumoniae* | OXA-232 | ≦1 | 2 | 8 | 16 | 128 | >128 | >256/4 | 256/128 | ≦0.5/4 | >64 | 1 | 1 | >32 | >128 | >128 |
| E109-1 | *K. pneumoniae* | OXA-232 | 64 | 64 | >128 | 64 | 64 | >128 | >256/4 | >256/128 | ≦0.5/4 | >64 | ≦0.5 | 0.5 | >32 | >128 | >128 |
| E104-1 | *K. pneumoniae* | OXA-232 | ≦1 | 2 | 8 | 16 | 128 | >128 | >256/4 | 256/128 | ≦0.5/4 | >64 | 1 | 0.5 | >32 | >128 | >128 |
| G412-2 | *K. pneumoniae* | OXA-232 | 2 | 2 | 8 | 64 | 64 | >128 | >256/4 | 256/128 | ≦0.5/4 | >64 | 1 | 1 | >32 | >128 | >128 |
| H415-2 | *K. pneumoniae* | OXA-232 | 8 | 32 | >128 | 64 | 128 | >128 | >256/4 | >256/128 | 1/4 | >64 | ≦0.5 | 1 | >32 | >128 | >128 |
| H415-1 | *K. pneumoniae* | OXA-232 | 64 | 16 | 64 | 16 | >128 | >128 | >256/4 | >256/128 | 1/4 | >64 | 2 | ≦0.25 | >32 | >128 | >128 |
| E111-1 | *K. pneumoniae* | OXA-232 | 64 | 4 | 32 | 16 | 128 | >128 | >256/4 | 256/128 | 1/4 | >64 | 2 | 1 | >32 | >128 | >128 |
| G415-1 | *K. pneumoniae* | OXA-232 | ≦1 | 4 | 4 | 4 | 32 | >128 | >256/4 | 128/64 | ≦0.5/4 | >64 | ≦0.5 | 1 | >32 | >128 | 128 |
| H420-4 | *K. pneumoniae* | OXA-232 | 16 | 32 | >128 | 64 | 64 | >128 | >256/4 | >256/128 | 1/4 | >64 | ≦0.5 | 1 | >32 | >128 | >128 |
| R250 | *K. pneumoniae* | OXA-232 | ≤1 | 2 | 16 | 16 | 64 | ＞128 | ＞256/4 | 128/64 | ≤0.5/4 | 64 | ≤0.5 | 1 | ＞32 | ＞128 | 128 |
| R256-2 | *K. pneumoniae* | OXA-232 | ≤1 | 2 | 16 | 16 | 64 | ＞128 | ＞256/4 | 128/64 | ≤0.5/4 | 64 | ≤0.5 | 1 | ＞32 | ＞128 | 128 |
| T238-1 | *K. pneumoniae* | OXA-232 | ≤1 | 2 | 16 | 16 | 64 | ＞128 | ＞256/4 | 128/64 | ≤0.5/4 | ＞64 | ≤0.5 | 2 | ＞32 | ＞128 | 128 |
| T238-2 | *K. pneumoniae* | OXA-232 | ≤1 | 2 | 16 | 16 | 64 | ＞128 | ＞256/4 | 128/64 | ≤0.5/4 | ＞64 | ≤0.5 | 2 | ＞32 | ＞128 | 128 |
| T256 | *K. pneumoniae* | OXA-232 | ≤1 | ≤2 | 16 | 16 | 64 | ＞128 | ＞256/4 | 128/64 | ≤0.5/4 | ＞64 | ≤0.5 | 1 | ＞32 | ＞128 | 128 |
| CYW-1 | *K. pneumoniae* | OXA-232 | 4 | 16 | 128 | 32 | 128 | ＞128 | ＞256/4 | 256/128 | ≤0.5/4 | ＞64 | 1 | ≤0.25 | ＞32 | ＞128 | ＞128 |
| R176-1 | *K. pneumoniae* | OXA-232 | 4 | 16 | 64 | 32 | 128 | ＞128 | ＞256/4 | >256/128 | ≤0.5/4 | ＞64 | ≤0.5 | ≤0.25 | 32 | ＞128 | ＞128 |
| R184-2 | *K. pneumoniae* | OXA-232 | 4 | 16 | 64 | 32 | 128 | ＞128 | ＞256/4 | >256/128 | ≤0.5/4 | ＞64 | ≤0.5 | ≤0.25 | 32 | ＞128 | ＞128 |
| R607-1 | *K. pneumoniae* | OXA-232 | ≤1 | 4 | 8 | 16 | 128 | ＞128 | ＞256/4 | 256/128 | 1/4 | ＞64 | ≤0.5 | ≤0.25 | 32 | ＞128 | ＞128 |
| R610-1 | *K. pneumoniae* | OXA-232 | ≤1 | 4 | 8 | 16 | 128 | ＞128 | ＞256/4 | 256/128 | 1/4 | ＞64 | ≤0.5 | ≤0.25 | 32 | ＞128 | ＞128 |
| R624-1 | *K. pneumoniae* | OXA-232 | ≤1 | ≤1 | 4 | 4 | 128 | ＞128 | ＞256/4 | 128/64 | ≤0.5/4 | ＞64 | 1 | ≤0.25 | 16 | ≤4 | ＞128 |
| K210042 | *K. pneumoniae* | OXA-232 | ≤1 | 2 | 64 | 16 | 64 | ＞128 | ＞256/4 | 256/128 | ≤0.5/4 | ＞64 | ≤0.5 | ≤0.25 | ＞32 | ＞128 | 64 |
| K210037 | *K. pneumoniae* | OXA-232 | ≤1 | 2 | 64 | 16 | 64 | ＞128 | ＞256/4 | 256/128 | ≤0.5/4 | ＞64 | 1 | ≤0.25 | ＞32 | ＞128 | 32 |
| K210038 | *K. pneumoniae* | OXA-232 | ≤1 | 4 | 64 | 16 | 64 | ＞128 | ＞256/4 | 256/128 | ≤0.5/4 | ＞64 | ≤0.5 | 0.5 | ＞32 | ＞128 | 64 |
| K210032 | *K. pneumoniae* | OXA-232 | ≤1 | 4 | 64 | 16 | 64 | ＞128 | ＞256/4 | 256/128 | ≤0.5/4 | ＞64 | ≤0.5 | 0.5 | ＞32 | ＞128 | 128 |
| K210025 | *K. pneumoniae* | OXA-232 | 2 | 4 | 64 | 32 | 64 | ＞128 | ＞256/4 | 256/128 | ≤0.5/4 | ＞64 | ≤0.5 | 1 | ＞32 | ＞128 | 64 |
| K210044 | *K. pneumoniae* | OXA-232 | 32 | 64 | ＞128 | 64 | 32 | ＞128 | ＞256/4 | 256/128 | ＜0.5/4 | ＞64 | ＞8 | ＞4 | ＞32 | ＞128 | 32 |
| K210024 | *K. pneumoniae* | OXA-232 | ≤1 | 4 | 64 | 32 | ＞128 | ＞128 | ＞256/4 | ＞256/128 | ≤0.5/4 | ＞64 | 1 | ≤0.25 | ＞32 | ＞128 | 128 |
| K210022 | *K. pneumoniae* | OXA-232 | ≤1 | 4 | 64 | 16 | 64 | ＞128 | ＞256/4 | 256/128 | ≤0.5/4 | ＞64 | 1 | ≤0.25 | ＞32 | ＞128 | 64 |
| E210065 | *E. coli* | OXA-232 | ≤1 | ≤1 | ≤2 | ≤2 | ≤2 | ≤4 | 256/4 | 32/16 | ≤0.5/4 | ≤4 | 1 | ≤0.25 | ≤1 | ≤4 | ≤4 |
| K210065 | *K. pneumoniae* | OXA-181 | 16 | 16 | ＞128 | 64 | ≤2 | ≤4 | 64/4 | 32/16 | ≤0.5/4 | ≤4 | 1 | 2 | 8 | ≤4 | ≤4 |
| K210053 | *K. pneumoniae* | OXA-232 | ≤1 | 4 | 32 | 16 | 64 | ＞128 | ＞256/4 | 256/128 | ≤0.5/4 | ＞64 | 1 | ≤0.25 | ＞32 | ＞128 | 64 |
| K210057 | *K. pneumoniae* | OXA-232 | ≤1 | 2 | 64 | 32 | 128 | ＞128 | ＞256/4 | 256/128 | ≤0.5/4 | ＞64 | 1 | ≤0.25 | ＞32 | ＞128 | 32 |
| K210051 | *K. pneumoniae* | OXA-232 | ≤1 | 2 | 64 | 32 | 64 | ＞128 | ＞256/4 | 256/128 | ≤0.5/4 | ＞64 | 1 | ≤0.25 | ＞32 | ＞128 | 32 |
| K210063 | *K. pneumoniae* | OXA-232 | ≤1 | 4 | 32 | 16 | 64 | ＞128 | ＞256/4 | 256/128 | ≤0.5/4 | ＞64 | 1 | ≤0.25 | ＞32 | ＞128 | 64 |
| K210005 | *K. pneumoniae* | OXA-232 | 64 | 64 | ＞128 | 64 | 64 | ＞128 | ＞256/4 | 256/128 | ≤0.5/4 | ＞64 | ≤0.5 | 0.5 | ＞32 | ＞128 | 128 |
| K210003 | *K. pneumoniae* | OXA-232 | 2 | 4 | 64 | 16 | 64 | ＞128 | ＞256/4 | 256/128 | ≤0.5/4 | ＞64 | 1 | ≤0.25 | ＞32 | ＞128 | 64 |
| K210006 | *K. pneumoniae* | OXA-232 | ≤1 | 4 | 64 | 16 | 64 | ＞128 | ＞256/4 | 256/128 | ≤0.5/4 | ＞64 | ≤0.5 | 0.5 | ＞32 | ＞128 | 64 |
| K210008 | *K. pneumoniae* | OXA-232 | ≤1 | 2 | 64 | 32 | 64 | ＞128 | ＞256/4 | 256/128 | ≤0.5/4 | ＞64 | ≤0.5 | 0.5 | ＞32 | ＞128 | 64 |
| K210010 | *K. pneumoniae* | OXA-232 | 2 | 4 | 64 | 32 | 64 | ＞128 | ＞256/4 | 256/128 | ≤0.5/4 | ＞64 | 1 | 0.5 | ＞32 | ＞128 | 64 |
| K210028 | *K. pneumoniae* | OXA-232 | 8 | 16 | 128 | 32 | 128 | ＞128 | ＞256/4 | 256/128 | ≤0.5/4 | ＞64 | ≤0.5 | 0.5 | 32 | ＞128 | ＞128 |
| K210013 | *K. pneumoniae* | OXA-232 | 2 | 8 | 128 | 32 | 64 | ＞128 | ＞256/4 | 256/128 | ≤0.5/4 | ＞64 | 1 | 1 | ＞32 | ＞128 | 32 |
| E210064 | *E. coli* | OXA-232 | ≤1 | ≤1 | ≤2 | ≤2 | ≤2 | ≤4 | 128/4 | 32/16 | ≤0.5/4 | ≤4 | 1 | ≤0.25 | ≤1 | ≤4 | ≤4 |
| K210017 | *K. pneumoniae* | OXA-232 | ≤1 | 4 | 64 | 32 | 128 | ＞128 | ＞256/4 | 256/128 | ≤0.5/4 | ＞64 | 1 | 0.5 | ≤1 | ≤4 | 32 |
| K210062 | *K. pneumoniae* | OXA-232 | ≤1 | 2 | 32 | 16 | 32 | ＞128 | ＞256/4 | 256/128 | ≤0.5/4 | ＞64 | 1 | 0.5 | ≤1 | ≤4 | 32 |
| K210060 | *K. pneumoniae* | OXA-232 | 4 | 8 | 64 | 32 | 64 | ＞128 | ＞256/4 | ＞256/128 | ≤0.5/4 | ＞64 | ≤0.5 | 0.5 | ＞32 | ＞128 | 32 |
| K210061 | *K. pneumoniae* | OXA-232 | ≤1 | 4 | 64 | 16 | 64 | ＞128 | ＞256/4 | 256/128 | ≤0.5/4 | ＞64 | 2 | 0.5 | ＞32 | ＞128 | 128 |
| K210052 | *K. pneumoniae* | OXA-232 | 2 | 4 | 64 | 32 | 128 | ＞128 | ＞256/4 | ＞256/128 | ≤0.5/4 | ＞64 | 1 | ≤0.25 | ＞32 | ＞128 | 64 |
| K210055 | *K. pneumoniae* | OXA-232 | ≤1 | 4 | 64 | 16 | 128 | ＞128 | ＞256/4 | 256/128 | ≤0.5/4 | ＞64 | 1 | ≤0.25 | ＞32 | ＞128 | 128 |
| K210059 | *K. pneumoniae* | OXA-232 | 128 | 64 | ＞128 | 64 | 64 | ＞128 | ＞256/4 | ＞256/128 | ≤0.5/4 | ＞64 | 1 | ≤0.25 | ＞32 | ＞128 | 64 |
| K210058 | *K. pneumoniae* | OXA-232 | 2 | 8 | 64 | 16 | 64 | ＞128 | ＞256/4 | 256/128 | ≤0.5/4 | ＞64 | 1 | ≤0.25 | ＞32 | ＞128 | 64 |
| K210056 | *K. pneumoniae* | OXA-232 | ≤1 | 8 | 32 | 16 | 64 | ＞128 | ＞256/4 | 256/128 | ≤0.5/4 | ＞64 | 1 | ≤0.25 | ＞32 | ＞128 | 64 |
| K210054 | *K. pneumoniae* | OXA-232 | 128 | 64 | ＞128 | 64 | 64 | ＞128 | ＞256/4 | ＞256/128 | ≤0.5/4 | ＞64 | 1 | ≤0.25 | ＞32 | ＞128 | 64 |
| K210064 | *K. pneumoniae* | OXA-232 | ≤1 | 4 | ＞128 | 16 | 32 | ＞128 | ＞256/4 | 256/128 | ≤0.5/4 | ＞64 | 1 | ≤0.25 | ＞32 | ＞128 | 64 |
| K210184 | *K. pneumoniae* | KPC-2 OXA-232 | 4 | 8 | 16 | ≤2 | 16 | 64 | ＞256/4 | 128/64 | ≤0.5/4 | 16 | ≤ | ≤0.25 | ≤1 | ≤4 | ＞128 |
| K210180 | *K. pneumoniae* | OXA-232 | ≤1 | ≤1 | 16 | 16 | 128 | ＞128 | ＞256/4 | 128/64 | ≤0.5/4 | ＞64 | 1 | 2 | ＞32 | ＞128 | ＞128 |
| K210185 | *K. pneumoniae* | OXA-232 | ≤1 | ≤1 | 16 | 8 | ＞128 | ＞128 | ＞256/4 | 128/64 | ≤0.5/4 | ＞64 | ≤0.5 | 1 | ＞32 | ＞128 | ＞128 |
| K210187 | *K. pneumoniae* | OXA-232 | 2 | 2 | 128 | 32 | 128 | ＞128 | ＞256/4 | 128/64 | ≤0.5/4 | ＞64 | 1 | ≤0.25 | ＞32 | ＞128 | 128 |
| K210182 | *K. pneumoniae* | OXA-232 | 2 | 4 | 32 | 32 | ＞128 | ＞128 | ＞256/4 | 256/128 | 1/4 | ＞64 | 1 | ≤0.25 | 32 | ＞128 | ＞128 |
| K210183 | *K. pneumoniae* | OXA-232 | ≤1 | ≤1 | 16 | 32 | 128 | ＞128 | ＞256/4 | 128/64 | ≤0.5/4 | ＞64 | 1 | 1 | ＞32 | ＞128 | 128 |
| K210186 | *K. pneumoniae* | OXA-232 | ≤1 | ≤1 | 32 | 32 | 128 | ＞128 | ＞256/4 | 128/64 | ≤0.5/4 | ＞64 | ≤ | 1 | ＞32 | ＞128 | 128 |
| K210181 | *K. pneumoniae* | OXA-232 | 16 | 32 | ＞128 | 64 | ＞128 | ＞128 | ＞256/4 | 256/128 | ≤0.5/4 | ＞64 | 1 | ≤0.25 | ＞32 | ≤4 | ＞128 |
| K210188 | *K. pneumoniae* | OXA-232 | 2 | 8 | 64 | 64 | 128 | ＞128 | ＞256/4 | 128/64 | ≤0.5/4 | ＞64 | ≤ | 4 | ＞32 | ＞128 | ＞128 |
| K210047 | *K. pneumoniae* | OXA-232 | 2 | 4 | 32 | 32 | 64 | ＞128 | ＞256/4 | 256/128 | ＜0.5/4 | ＞64 | ≤ | 1 | ＞32 | ＞128 | 64 |
| K210048 | *K. pneumoniae* | OXA-232 | ≤1 | 4 | 32 | 16 | 128 | ＞128 | ＞256/4 | 256/128 | ≤0.5/4 | ＞64 | ＞8 | 0.5 | ＞32 | ＞128 | ＞128 |
| K210049 | *K. pneumoniae* | OXA-232 | 8 | 32 | 64 | 32 | 64 | ＞128 | ≤ | 32/16 | ≤0.5/4 | ＞64 | 1 | 2 | ＞32 | ＞128 | 32 |
| K210050 | *K. pneumoniae* | OXA-232 | 64 | 64 | ＞128 | 64 | 64 | ＞128 | ＞256/4 | ＞256/128 | ≤0.5/4 | ＞64 | 1 | 0.5 | ＞32 | ＞128 | 128 |
| K210259 | *K. pneumoniae* | OXA-232 | 2 | 4 | 32 | 16 | 64 | ＞128 | ＞256/4 | 256/128 | ≤0.5/4 | 64 | 8 | 1 | ＞32 | ＞128 | 128 |
| K210261 | *K. pneumoniae* | OXA-232 | 2 | 8 | 32 | 32 | 32 | ＞128 | ＞256/4 | ＞256/128 | ≤0.5/4 | 64 | ≤ | 0.5 | 16 | ＞128 | 128 |
| K210263 | *K. pneumoniae* | OXA-232 | 4 | 8 | 64 | 32 | ＞128 | ＞128 | ＞256/4 | ＞ | ≤0.5/4 | ＞64 | 1 | 0.5 | ＞32 | ＞128 | ＞128 |
| K210265 | *K. pneumoniae* | OXA-232 | 2 | 8 | 32 | 16 | 64 | ＞128 | ＞256/4 | 256/128 | ≤0.5/4 | ＞64 | 8 | 1 | ＞32 | ＞128 | 128 |
| K210269 | *K. pneumoniae* | OXA-232 | 2 | 4 | 32 | 16 | 32 | ＞128 | ＞256/4 | ＞256/128 | ＜0.5/4 | ＞64 | ＜0.5 | 1 | ＞32 | ＞128 | 64 |
| K210276 | *K. pneumoniae* | OXA-232 | 2 | 4 | 32 | 16 | 64 | ＞128 | ＞256/4 | 256/128 | ≤0.5/4 | 64 | ＞8 | 1 | ＞32 | ＞128 | 64 |
| K210279 | *K. pneumoniae* | OXA-232 | 2 | 8 | 32 | 16 | 64 | ＞128 | ＞256/4 | 256/128 | ≤0.5/4 | ＞64 | ＞8 | 1 | ＞32 | ＞128 | 128 |
| K210281 | *K. pneumoniae* | OXA-232 | ≤1 | 4 | 32 | 32 | 64 | ＞128 | ＞256/4 | ＞256/128 | ≤0.5/4 | 64 | ≤ | 0.5 | 16 | ＞128 | 128 |
| K210282 | *K. pneumoniae* | OXA-232 | ≤1 | 4 | 32 | 16 | 32 | ＞128 | ＞256/4 | ＞256/128 | ≤0.5/4 | 64 | ≤ | 0.5 | 16 | ＞128 | 64 |
| K210284 | *K. pneumoniae* | NDM-1;OXA-232 | 32 | 64 | ＞128 | ＞128 | ＞128 | ＞128 | ＞256/4 | ＞256/128 | ＞64/4 | ＞64 | ≤ | 1 | 32 | ＞128 | 128 |
| K210309 | *K. pneumoniae* | OXA-232 | 32 | 32 | >128 | 64 | 128 | >128 | >256/4 | 256/128 | ≤0.5/4 | >64 | 1 | 2 | >32 | >128 | >128 |
| K210317 | *K. pneumoniae* | OXA-232 | 2 | 4 | 32 | 32 | 128 | >128 | >256/4 | 128/64 | ≤0.5/4 | >64 | 1 | 2 | >32 | >128 | >128 |
| K210327 | *K. pneumoniae* | OXA-232 | ≤1 | ≤1 | 8 | 8 | 128 | >128 | >256/4 | 256/128 | ≤0.5/4 | >64 | ≤0.5 | 0.5 | >32 | >128 | >128 |
| K210334 | *K. pneumoniae* | OXA-232 | ≤1 | 4 | 32 | 32 | 32 | >128 | >256/4 | 128/64 | ≤0.5/4 | >64 | ≤0.5 | 4 | >32 | >128 | 128 |
| TC-122 | EC600 | NDM-1 | 8 | 16 | 16 | 64 | ＞128 | 128 | 256/4 | 256/128 | ＞64/4 | 64 | ＜0.5 | ＜0.25 | ＜1 | ＜4 | ＜4 |
| TC-40 | EC600 | KPC-2 | 4 | 4 | 16 | 16 | 16 | 128 | ＞256/4 | 128/64 | ＜0.5/4 | 32 | ＜0.5 | ＜0.25 | 2 | ＜4 | 128 |

Table S3. Antimicrobial susceptibility profiles of OXA-48-like-producing strains and transconjugants.
